# Early Word Order Usage in Preschool Mandarin-Speaking Typical Children and Children With Autism Spectrum Disorder: Influences of Caregiver Input?

**DOI:** 10.3389/fpsyg.2021.766133

**Published:** 2022-01-06

**Authors:** Ying Alice Xu, Letitia R. Naigles, Yi Esther Su

**Affiliations:** ^1^Child Language Lab, School of Foreign Languages, Central South University, Changsha, China; ^2^Department of Psychological Sciences, University of Connecticut, Storrs, CT, United States

**Keywords:** word order, mandarin, preschool, typical children, children with ASD, caregiver input

## Abstract

This study explores the emergence and productivity of word order usage in Mandarin-speaking typically-developing (TD) children and children with autism spectrum disorder (ASD), and examines how this emergence relates to frequency of use in caregiver input. Forty-two caregiver-child dyads participated in video-recorded 30-min semi-structured play sessions. Eleven children with ASD were matched with 10 20-month-old TD children and another 11 children with ASD were matched with 10 26-month-old TD children, on expressive language. We report four major findings: (1) Preschool Mandarin-speaking children with ASD produced word order structures with pervasive ellipsis at similar rates to language-matched TD children, but also displayed differences from TD children in their usage of SVt and VtO frames; (2) Grammatical productivity was observed in both TD children and children with ASD; moreover, children with ASD with higher expressive language produced less stereotyped language; (3) Both TD children and children with ASD heard a range of word orders in their caregivers’ input, with TD children’s input greater in amount and complexity; however, caregivers of both groups also showed no age/language-related changes in word order usage; (4) Few word-order-specific correlations emerged between caregivers and their children; however, strong correlations were observed for mean length of utterances (MLU) for both groups: Caregivers who produced longer/more complex utterances had children who did the same. Taken together, it seems that despite their pragmatic deficits, the early grammatical knowledge of word order in Mandarin-exposed children with ASD is well preserved and in general follows the typical developmental pattern. Moreover, caregiver input is broadly rather than finely tuned to the linguistic development of TD children and children with ASD, and plays a more important role in children’s general syntactic development than in specific word order acquisition. Thus, early word order usage in preschool Mandarin-speaking TD children and children with ASD may be influenced by both caregiver input and child abilities.

## Introduction

Caregiver input and child abilities have both been implicated in children’s successful acquisition of the grammar of their native language (Crain and Pietroski, 2001; Valian, 2009; Fusaroli et al., 2019; Rowe and Snow, 2020). Word order is the foundation of grammar in many languages. While Mandarin sentences canonically follow SVO order like English, Mandarin also allows pervasive noun phrase (NP) ellipsis and supports various non-canonical word orders. For example, omissions of subject [Verb Object (VO); e.g., “chi1 bing3gan1”/Eat cookies] or object [Subject Verb (SV); e.g., “wo3 chi1”/I eat], or omissions of even both (V; e.g., “chi1”/Eat) are quite common in Mandarin (Lee and Naigles, 2005), and such frequent omissions of subject and object further complicate Mandarin acquisition. Previous research on word order production by Mandarin learners has indicated early acquisition (Erbaugh, 1982; Wang et al., 1992; Yeh, 2015; Fan and Song, 2016); however, questions remain concerning the facilitating roles of caregiver input and child abilities. Studying the effect of caregiver input on children’s word order acquisition, as well as including children with autism spectrum disorder (ASD) who have impairments in social interaction and communication as a comparison group, may help address these questions.

Children with ASD are known to have impairments in social interaction and communications (American Psychiatric Association, 2013), and such social challenges may impact Mandarin word order development directly, as learning to use SV and VO frames requires understanding that the “missing” NP is part of the speaker’s and hearer’s shared perspective (Su and Naigles, 2019). Moreover, these social challenges may result in fewer overt social cues to their caregivers. This lack of affective reciprocity may have negative impacts on caregivers’ own communicative styles (Kim and Mahoney, 2004). Caregivers of children with ASD may believe that their children with ASD do not benefit from their home linguistic environment. However, recent research has pointed to a strong and positive role of caregiver input in language acquisition of English-learning children with ASD (Naigles, 2013; Goodwin et al., 2015; Nadig and Bang, 2017; Fusaroli et al., 2019; Bottema-Beutel and Kim, 2020). The current study breaks new ground in investigating the emergence and productivity of elided and non-canonical word orders in Mandarin, by comparing this emergence in TD children and children with ASD, and by examining how this emergence relates to frequency of use in concurrent caregiver speech.

### TD Children’s Word Order Acquisition in Mandarin Chinese

Word order is a fundamental property that constitutes the basic syntactic structure in many languages. English is a strict SVO word order language (Valian, 1991; Wang et al., 1992) with obligatory overt subjects and objects. In contrast, while Mandarin’s canonical word order is SVO, it allows elided SVO-related word orders, such as SVi, SVt, VtO, as well as various non-canonical word orders, such as OV, *VS*, OSV, SOV, plus the Ba and Bei constructions (Lü, 2002; See Table 1 for definitions and examples of each order). Studies of TD children’s development of Mandarin have revealed early usage of the canonical word orders. For example, Erbaugh (1982) recorded and analyzed four child Mandarin learners’ spontaneous speech from 2 to 3;5 years of age, and found that their early two-word combinations included both SV and VO. The VO order was somewhat the more frequent, presumably because the subjects were nearly always the children or their hearers, thus shared in common ground and so more likely to be omitted. The children were consistently producing full SVO sentences by age 3. Fan and Song (2016) explored the early acquisition of SVO-related word orders in a single child from 1;0 to 2;6, reporting that all orders were produced before 1;6. Yeh (2015) also found this developmental pattern in her analyses of the Zhou (2001) corpus, which included 10 Mandarin-learning children’s speech at each of 14, 20, 26, and 32 months of age, showing that the children produced SVi, SVt, and VtO word orders by the age of 20 months and SVtO at 26 months old. Elicited production studies have yielded similar findings, with 2-year-old producing more VO than SV utterances, and children reaching adult levels of overt subject and object production by age 4 (Wang et al., 1992; Kim, 2000). Finally, comprehension studies using intermodal preferential looking (IPL) have also reported Mandarin learners’ ability to process reversible SVO sentences accurately by 2-to-3 years of age (Candan et al., 2012; Su and Naigles, 2019).

**Table 1:** Word order frames.

Word order	Pinyin	Translation
**Verb alone**		
Vi^*^	Zuo4 xia4	Sit down
Vt^*^	Kan4	See
Va^*^	Zhen1 shuai4	Very handsome
**Canonical**		
SVi^*^	Ni3 zuo4	You sit (down)
SVt^*^	Ni3 kan4	You see
SVa	Wa2wa e4 le	The doll (is) hungry
SVtO^*^	Wo3 yao4 qi4qiu2	I want balloon
VtO^*^	Chi1 bing3gan1	Eat cookie
(S)P(O/N)V(O)	Ni3 gei3 ta1 wei4 niu2nai3	You feed her milk
**Non-Canonical**		
(S)Ba(O)V	Ni3 ba3 ji1mu4 bai3 zhe4li3	You Ba the block put here
OV^*^	Qi4che1 fang4 na4li3	The car put there
*VS*	Zhe4li3 lai2 le ge4 xiao3 dong4wu4	Here comes a little animal
OSV	Zhe4ge wo3men bu4 wan2 le	This we do not play
SOV	Ni3 zhe4ge4 ye3 hui4 wan2	You this also know how to play
(O)Bei(S)V	Gong1jiao1che1 hui4 bei4 ya1 bian3	The bus will Bei (be) crush(ed)
**Copular**		
(S)Pnom^*^	Zhe4 shi4 shen2me yan2se4	What is this color
(S)Padj	Zhe4 liang3ge4 shi4 yi2yang4 de	These two are the same
(S)PV(O)	Zhe4 shi4 ting1 ge1 de	This is for listening music
(S)zai(Loc)^*^	Mao2mao2chong2 zai4 zhe4li3	The caterpillar is here
(Loc)You(NP)^*^	Dai4zi3 li3 you3 shen2me dong1xi	What (thing) is there in the bag
(Loc)Be(NP)	Dai4zi3 li3 shi4 shen2me dong1xi	What (thing) is in the bag
**Topicalized**		
TSV(O)	Liang3ge4 he2zi ni3 yao4 na3 yi2ge4	As for two boxes, which one do you want
**V-no-V**		
V-no-V	Ni3 yao4 bu2 yao4 bing3gan1	Do you want cookie or not
**Multiverb** ^*^		
(S)V1(O)V2(O)	Wo3men lai2 dui1 ji1mu4	Let us build blocks
(S)V1V2(O)	Ni3 xi3huan1 chi1 bing3gan1 ma1	Do you like eating cookies
(S1)V1(O)(S2)V2(O)	Ni3 kan4 ma1ma zen3me die2 de	You see how does mom build
(S)V1NV2(O)	Ni3 yao4 ma1ma na2 ma1	Do you want mom to take (it)
(S)V1(O)PNV2	Ni3 da1 ji1mu4 gei3 wo3 kan4 xia4	Let me see you building blocks
Combination	Wo3men zai4 zhao3 xiao3peng2you3 jin4lai2 zuo4	Let us find more kids to come in and have a sit

Va, adjectival verb; SVi, subject + intransitive verb; SVt, subject + transitive verb; VtO, transitive verb + object; (S)P(O/N)V(O), subject + preceding preposition(coverb) + object/noun phrase + verb + object; (S)Pnom, subject + copula + nominal predicate; and (Loc)You(NP), location + you3 + noun phrase.

* indicates those used in the current analyses.

The emergence of non-canonical word orders has been studied less frequently. For example, Fan and Song (2016) reported that the OV order was produced by their participant approximately 6 months after SVO, and Yeh (2015) observed children producing the frames (S)Pnom, (Loc)You(NP), and OV by the age of 32 months. Thus, more studies are needed that focus on the development of non-SVO word orders in Mandarin Chinese. A second gap in the literature on early word order development in Mandarin involves investigations of the *productivity* of Mandarin learners’ early speech, which may be related to children’s innate linguistic abilities (Chomsky, 1959). That is, early multi-word utterances might be produced as full or partial repetitions of preceding input or as formulaic utterances (Wray, 2008), neither of which would be considered productive. Alternatively, these utterances might be produced in a combinatory way through the operation of grammatical rules on representations and understood analytically, hence productive (Perkins, 1999). Example 1 illustrates the distinction between children’s productive and non-productive utterances.

*Example 1*

Mom: “Zi4ji3 da3kai1 gai4zi”/Open the lid (by yourself)

Child A: “Ba4ba da3kai1 zhe4ge”/Dad opens this. (productive)

Child B: “(Zi4ji3) da3kai1 gai4zi”/Open the lid (by yourself; non-productive).

The current study will address both of these gaps, as we investigate the emergence of both canonical and non-canonical word orders in child Mandarin, as well as assess the productivity of the children’s most frequently used Mandarin word orders.

### Early Word Order Acquisition in Children With ASD

Very few published studies have described early word order acquisition in Mandarin-speaking children with ASD. Zhou et al. (2017) used picture selection tasks to test sentence comprehension of 80 4-to-5-year-old Mandarin-speaking children with high-functioning autism (HFA). Children with HFA correctly chose pictures corresponding to sentences in SVO order. Moreover, Zhou et al. (2017) also gave sentences with the morphosyntactic markers Ba and Bei, forming the (S)Ba(O)V and (O)Bei(S)V frames, to test whether children with HFA were able to use the information coded in the markers vs. just relied on the word order to promote sentence comprehension. The results found that children with HFA could effectively use both word order and morphosyntactic marker cues in sentence comprehension. But they relied significantly more on word order when there were conflicts between these two linguistic cues, compared to age-matched TD children. Using IPL, Su and Naigles (2019) reported intact – if somewhat slower – SVO word order processing in 70 2-to5-year-old (*M* = 49.57 m) Mandarin-speaking children with ASD, compared with TD controls, further supporting that the core grammatical structures may be well-preserved in children with ASD across languages. Even some minimally verbal children with ASD exhibited SVO comprehension despite their profoundly impaired expressive language skills.

So far, there are no published studies reporting production data from language samples that explored early word order acquisition in Mandarin-speaking children with ASD, which is another gap that this research intends to fill. If early Mandarin grammar production tracks the TD case, as described above, then children with ASD would produce SV and VO frames first with VO the most frequent, followed by SVO and then eventually OV orders (Erbaugh, 1982; Wang et al., 1992; Kim, 2000; Yeh, 2015; Fan and Song, 2016). However, it is also possible that Mandarin child learners with ASD, because of their difficulties in understanding and participating in social interactions, might display a different developmental pattern. This is because in a null argument language like Mandarin, common ground is needed for speakers and listeners to recover dropped nouns in SVt, VtO, and V-only utterances (Su and Naigles, 2019). For example, “chi1” (eat) can only be produced as a V-only utterance when the subject [e.g., “wo3”(I)] and object [e.g., “bing3gan1”(cookie)] have already been referenced between speaker and listener. One way to establish this common ground is through joint attention, which has been demonstrated to be challenging to be established and maintained for children with ASD (Mundy, 2018; Su et al., 2018). Therefore, it is possible that young Mandarin learners with ASD might acquire full SVO frames first, but not SVt, VtO, or V-only utterances.

Additionally, previous research – and clinical assessments – has frequently suggested that English-speaking children with ASD produce significantly more formulas and significantly less productive language than TD children (Tager-Flusberg and Calkins, 1990). However, Van Lancker Sidtis (2012) has argued that formulaic language might not be a characteristic of the entire ASD spectrum; she observed that adults who were at the lower functioning end of the autism spectrum produced formulaic expressions almost exclusively, while those toward the higher-functioning end of the autism spectrum communicated with almost no formulaic expressions. Nothing is known about productivity vs. formulaic language in Mandarin learning children with ASD, and the current study aims to address this gap.

### Role of Caregiver Input/Talk in Early Grammatical Acquisition/Development

Many studies investigating the role of caregiver input in the early grammatical acquisition of English-speaking TD children have documented that children who hear longer and more diverse grammatical structures from their caregivers subsequently produce longer and more diverse grammatical structures, themselves (e.g., Huttenlocher et al., 2010; see Rowe and Snow, 2020, for a more comprehensive review). However, especially with specific complex syntactic structures, sheer frequency also appears to play a role. For example, children who heard stories including more passive sentences showed more frequent use of the passive voice several months later (Vasilyeva et al., 2006; Ambridge et al., 2015). And within conversations, caregivers tend to adapt their structural complexity to the language levels of their TD children (Rowe and Snow, 2020).

Somewhat surprisingly, similar findings have been observed with English-learning children with ASD. That is, given that the children with ASD have pragmatic and social deficits, maintaining conversations is a more difficult task for both child and caregiver, compared with TD children (Siller and Sigman, 2002). Children with ASD appear to be paying less overt attention to their caregivers’ input. However, caregivers of children with ASD apparently adjust their speech to fit their children’s language levels, as few differences have been found in mean length of utterances (MLU) or sentence complexity between caregiver of children with ASD vs. those of language-matched TD children (Cantwell et al., 1977; Wolchik and Harris, 1982; Konstantareas et al., 1988; Watson, 1998; Slaughter et al., 2007; Venuti et al., 2012; Bang and Nadig, 2015; Bottema-Beutel and Kim, 2020). Furthermore, caregivers producing utterances with longer MLUs had children with ASD who subsequently produced utterances with longer MLUs (Bang and Nadig, 2015; Fusaroli et al., 2019), and caregivers who produced fewer wh-questions with diverse verbs had children who showed poorer wh-question comprehension (Goodwin et al., 2015).

There has been very limited research on caregiver input influences on the word order development of Mandarin-speaking TD children. In the limit, of course, Mandarin learners acquire Mandarin because of their input, and language-specific grammatical production patterns can be observed early; for example, 2-year-old Mandarin learners produce more VtO and SVt utterances than 2-year-old English learners (Wang et al., 1992). However, Erbaugh (1982) found few relationships between caregiver frequency of use of SVO-related and non-SVO constructions (Ba and Bei constructions, OV, OSV, and SOV), and child frequency of use, in two mother-child dyads, except for the Ba construction. Considering 10 children at each of 14, 20, 26, and 32 months of age, Yeh (2015) found that high-frequency word orders in mothers’ speech (SVO-related word orders and copular constructions) tended to appear in children’s speech by the age of 26 months, while low-frequency word orders (non-SVO word orders) tended to appear later and less frequently. Within this dataset, greater frequency of use of the Ba construction by caregivers was not reflected in greater frequency of use by their children, although caregiver frequency effects were observed for five word orders [Vi, Vt, Va, SVtO and (S)Pnom utterances] at 20 and 26 months. Interestingly, none of these studies has found that caregivers of older children produced more diverse word orders than those of younger children, although diversity in word order use may not be a valid indicator of caregiver adaptation to children’s growing communicative competence in Mandarin (Erbaugh, 1982; Wang et al., 1992; Yeh, 2015).

So far, there is no published research concerning the effects of caregiver input on the word order acquisition of Mandarin-speaking children with ASD; the current study, therefore, breaks completely new ground in this area.

### Research Objectives and Hypotheses

In the current study, we examine the grammatical usage of 20 and 26-month-old Mandarin-exposed TD children, and of children with ASD who are language-matched, focusing on the word orders produced during caregiver-child interactions. These age groups were recruited because this is the transition period when children develop from producing one-word utterances to producing two- or multi-word stage (Brown, 1973; Erbaugh, 1982; Hoff, 2001; Yeh, 2015). Comparisons will be carried out within each diagnostic group of children but across ages to examine their word order development patterns, and between TD children and children with ASD to explore etiological differences in word order development. Moreover, the children’s productivity of the most frequent word order patterns will be assessed in both diagnostic groups. Caregiver speech will also be examined, for child age/language level differences and for diagnostic group differences, as well as to investigate when children’s word orders approach adult frequency and distributional patterns. Because of the concurrent study design, and the small number of dyads in this study (10–11/group), our focus will be on what we call “same-frame” correlations (i.e., did caregivers who produced word order X more frequently have children who also produced word order X more frequently?) as well as general correlations (i.e., did caregivers who produced more utterances/more complex utterances overall have children who also produced more utterance/more complex utterances, or more of any specific word order?).

Studies of Mandarin learners of word orders are needed because systematic research about word order acquisition of this language, which allows pervasive ellipsis of NPs and various types of word orders, is still very sparse. Better understanding of TD children’s Mandarin acquisition is also needed in order to discern the degree to which the acquisition by children with ASD is similar or different. For example, are the patterns of emergence of the SV, VO, and SVO orders, as well as the non-canonical orders, that are seen in TD children, also seen in children with ASD? Producing SVt and VtO frames implicates the ability to recover subjects and objects that are shared in common ground, shedding light on the extent to which discourse/pragmatic knowledge is required in acquiring grammatical structures. Moreover, investigation of children’s productivity with word order is necessary to deepen our understanding of child abilities in grammatical acquisition. Furthermore, studies investigating input/caregiver effects on Mandarin-speaking children with ASD’s development of grammar are needed because little is known about the extent to which grammatical development of children with ASD related to their caregiver input, and which aspects of input are most beneficial to young learners with ASD.

Based on the previous literature, our hypotheses are as follows:

1. Preschool Mandarin-learning TD learners will begin to produce multi-word sentences with subjects and objects during their 3rd year of life. Given their pragmatic deficits, children with ASD will produce these sentences at later “language ages” than TD children. Moreover, when matched with TD children in language, children with ASD are likely to produce more SVO word orders than SVt and VtO orders; for example, saying “wo3 chi1 bing3gan1” (I eat cookies) rather than “chi1” (eat) when being asked “ni3 chi1 bing3gan1 ma1?” (Do you eat cookies?).
2. For TD children, SVO-related word orders will reach adult frequency level by the age of 26 months, whereas production of the non-SVO word orders will still be sparse. For children with ASD, SVO frames themselves will reach adult levels of frequency before the SVt and VtO frames; non-canonical word orders will again emerge later.
3. Typically-developing children will demonstrate productivity with their most frequent word orders whereas children with ASD will show much more formulaic or repetitive usage, at least within the age ranges studied here.
4. No age-related changes will be observed in the caregiver input for TD children; however, caregivers of children with ASD will speak differently to their children, depending on whether they are lower-verbal vs. higher-verbal. Moreover, the caregiver input of children with ASD will be lower in quantity and diversity than that of language-matched TD children.
5. Finally, caregiver input for TD children will correlate with the children’s output, both generally (e.g., caregiver MLU correlating with child MLU) and specifically (e.g., caregiver use of SVt correlating with child use of SVt). Fewer relationships will be observed within the dyads that include children with ASD, because their social and communication impairments will make them less responsive to caregiver input.

## Materials and Methods

### Participants

The final dyads included 20 TD children (10 in the 20-month-old group and 10 in the 26-month-old group) and 22 children with ASD matched on expressive language (11 in the 20 m-matched group, 11 in the 26 m-matched group), along with their caregivers. Most children were accompanied by their mother or father; however, three children with ASD were accompanied by their grandmothers as these were the primary caregivers. All children were Mandarin-exposed. Children with ASD were recruited from three autism training centers in Changsha, China. The diagnoses were ascertained by experienced child psychiatrists on the basis of the Diagnostic and Statistical Manual of Mental Disorders, Fourth Edition, Text Revision (DSM IV-TR; American Psychiatric Association, 2000). The diagnoses were also confirmed by the caregiver rating of the Chinese Autism Behavior Checklist (ABC; Yang et al., 1993), whose cut off score is 31. All the children with ASD had ABC scores ≥ 31 whereas all the TD children had ABC scores lower than 31. The TD group was recruited by word of mouth; targeting children aged 20 or 26 months who had, by caregiver report, no developmental delay or disorder. All the caregivers were asked to fill out the Putonghua Communicative Development Inventory (PCDI; Tardif et al., 2008), and each signed consent forms for participation at their visit. The mean age, ABC, and PCDI scores for each group are presented in Table 2; as the table shows, the TD and language-matched ASD groups differed on age and ABC scores, but not on PCDI scores.

**Table 2:** Participant characteristics.

	20 m TD (*n* = 10) Mean (*SD*)	20 m-matched ASD (*n* = 11) Mean (*SD*)	*t*	*p*	26 m TD (*n* = 10) Mean (*SD*)	26 m-matched ASD (*n* = 11) Mean (*SD*)	*t*	*p*
Age (months)	20.00 (0.0)	43.45 (5.7)	−13.609	<0.001	26.20 (0.6)	50.91 (8.0)	−10.202	<0.001
ABC	7.60 (7.5)	49.64 (14.6)	−8.379	<0.001	13.10 (9.4)	59.36 (23.1)	−6.119	<0.001
PCDI (words)	189.80 (182.8)	173.09 (108.9)	0.257	0.800	457.20 (153.0)	459.00 (92.3)	−0.032	0.975
Sex Ratio (M:F)	6:4	6:5			7:3	9:2		

ABC, Autism Behavior Checklist; PCDI, Putonghua Communicative Development Inventory.

### Standardized Measures

The ABC was designed to assess children’s autistic behaviors through a parent/teacher rating scale (Krug et al., 1980). The Chinese version of the ABC (Yang et al., 1993) was used as the diagnostic confirmation in this research. Children with scores of 31 or more were confirmed to be in the ASD group, and children with scores under 31 were confirmed to be in the TD group.

The PCDI (Tardif et al., 2008) is an adapted version of MacArthur-Bates Communicative Development Inventory. There are two forms of PCDI: PCDI – Words and Gestures (Infant Form) and PCDI – Words and Sentences (Toddler Form). The latter form was used in this study to measure children’s language production abilities through caregiver report (see also Su et al., 2018).

### Procedure

The dyads of children with ASD and their caregivers were video-recorded in a quiet room in their autism training center, while those of TD children and their caregivers were video-recorded in the Child Language Lab of Central South University in Changsha, China. Caregiver and child engaged in a 30-min semi-structured play session, whose format was based on the Screening Tool for Autism in 2-year-old (Stone et al., 2000; see also Tek et al., 2014; Fusaroli et al., 2019). Caregivers were encouraged to interact with their children in the way they normally did at home. Every pair of caregiver and child was provided with same or similar toys and books on the mat where they sat. The first 5 min of the session were designated as free play/warm up. For the next 15 min, caregivers were periodically handed cards that instructed them to play with particular items that the researchers had provided. For example, the child was asked to build a tower with blocks and then push it down; the caregiver was asked to blow up a balloon and let it go; and the caregiver and child were asked to read a book together, etc. The last 10 min were again designated as free play. The entire play session was recorded and later transcribed.

### Transcription and Coding

Both caregivers and children’s utterances were transcribed by undergraduates/graduates according to the conventions provided by the Codes for the Human Analysis of Transcripts (MacWhinney, 2000). All the transcripts were then reviewed and later coded by the first author. Any discrepancies with the transcription and problems with coding were discussed among the authors and resolved by consensus; no major discrepancies were found. Coding focused on the caregivers and children’s spontaneous speech, excluding memorized or routine phrases, such as social routines, songs, poems, nursery rhymes, and story lines in books. All utterances with one or more verbs were coded and assigned to one of the 24 word order frames listed in Table 1 (with examples), which were derived from the frames developed by Lee and Naigles (2005).

Formulaic utterances are sentences which are not newly created based on the operation of grammatical rules. In this study, formulaic utterances were coded according to Tager-Flusberg and Calkins (1990), and included (a) routines (including songs, games, social routines, book reading, commercials, etc.), (b) self-repetition, and (c) imitations of the interlocutor’s utterances. Within the category of routine, the occasions in which mother and child frequently engaged (e.g., dining, bathing, etc.) were also considered to be formulaic.

### Data Analysis

Because both children and caregivers varied in terms of total utterances produced, frequencies of word order were calculated as percentages of the total coded utterances and compared across groups. Because 12 of the 24 coded frames were used very rarely (under 2.0% of utterances), only 12 frames were analyzed for this study: Vi, Vt, Va, SVi, SVt, SVtO, VtO, OV, (S)Pnom, (S)zaiLoc, (Loc)You(NP), and Multiverb utterances (see frames with * in Table 1). The OV frame, while used quite infrequently, was included in our analyses because it was often the first non-canonical frame the children produced. Five sets of independent sample *t*-tests were used to compare these word order structures: (1) across ages/language levels to track the developmental pattern of both TD children and children with ASD; (2) between TD children and language-matched children with ASD to explore group differences in word order development; (3) between caregivers and children to discover which word orders had reached adult frequency levels; (4) between caregivers of the two age groups in the TD group and the two language groups in the ASD group to explore the age-/language-related differences in input; and (5) between caregivers of TD children and those of children with ASD to detect group-based input differences. Additional analyses related to child productivity are described below. Finally, correlations between caregivers’ and children’s usage of the same word orders were calculated to investigate whether frames that were used more frequently by caregivers were also those that were used more frequently by children.

## Results

Each comparison involved the number of total utterances, the frequency of usage of frames/word orders as a function of the number of total utterances (i.e., percent of usage of each frame/word order), and MLU. Twelve frames (Vi, Vt, Va, SVi, SVt, SVtO, VtO, OV, (S)Pnom, (S)zai(Loc), (Loc)You(NP), and Multiverb) were included in the analyses.

### Children’s Word Order Utterances

Table 3 shows the means and SDs of word order utterances for each child group, as well as the age/language-level comparisons for the TD and ASD groups. As the Table shows, the older 26 m TD children/more language-advanced children with ASD generally produced the multi-word frames a higher percentage of the time than the younger 20 m TD children/less language-advanced children with ASD, who generally produced a higher percentage of verb-only frames. These comparisons reached statistical significance for the SVt, Multiverb, OV, and (S)zai(Loc) word orders, total utterances, and MLU for the TD children, and for the VtO and (Loc)You(NP) word orders for the children with ASD.

**Table 3:** Between-group comparison of word order utterances (Children; percent of total utterances).

Word order	20 m TD Mean (*SD*)	26 m TD Mean (*SD*)	*t*	*p*	20 m-matched ASD Mean (*SD*)	26 m-matched ASD Mean (*SD*)	*t*	*p*
Vi	11.20 (11.3)	6.20 (5.3)	1.266	0.222	11.47 (10.7)	10.82 (6.6)	0.172	0.866
Vt	27.62 (22.8)	26.26 (15.2)	0.158	0.876	33.33 (28.8)	25.80 (13.7)	0.783	0.443
Va	5.69 (6.3)	3.71 (3.0)	0.900	0.380	9.18 (15.0)	2.93 (3.7)	1.346	0.205
SVi	2.04 (3.0)	4.58 (3.5)	−1.727	0.101	1.50 (2.6)	3.07 (3.3)	−1.226	0.234
**SVt**	**3.44 (4.9)**	**7.95 (1.9)**	**−2.714**	**0.019**	9.04 (19.3)	4.31 (3.8)	0.797	0.435
SVtO	2.08 (4.1)	5.33 (5.0)	−1.591	0.129	5.78 (11.9)	4.11 (3.8)	0.443	0.663
**VtO**	6.81 (7.4)	7.57 (4.5)	−0.278	0.785	**6.55 (5.9)**	**15.62 (4.5)**	**−4.018**	**0.001**
**OV**	**0.10 (0.3)**	**1.61 (1.4)**	**−3.244**	**0.009**	1.85 (2.5)	2.73 (3.2)	−0.722	0.478
(S)Pnom	9.85 (20.5)	6.63 (9.2)	0.453	0.656	7.91 (14.5)	7.90 (7.0)	0.001	0.999
**(S)zai(Loc)**	**0.75 (1.6)**	**2.87 (2.5)**	**−2.256**	**0.037**	0.29 (0.6)	2.26 (3.5)	−1.844	0.093
**(Loc)You(NP)**	6.23 (12.1)	8.81 (5.0)	−0.623	0.541	**0.88 (2.4)**	**3.42 (2.5)**	**−2.413**	**0.026**
**Multiverb**	**1.95 (3.2)**	**14.37 (9.5)**	**−3.905**	**0.002**	10.41 (9.5)	14.07 (6.9)	−1.043	0.309
**Total utterances**	**41.00 (43.7)**	**95.00 (39.0)**	**−2.916**	**0.009**	58.91 (64.5)	95.36 (83.8)	−1.144	0.266
**MLU**	**1.55 (0.5)**	**2.17 (0.5)**	**−2.703**	**0.015**	2.02 (0.7)	2.23 (0.5)	−0.844	0.409

Data in bold refer to comparisons of word order usages, which reached significance.

We also compared the percent of total utterances with which children produced each frame between the TD and ASD groups within each age/language level. Given that the 20 m TD and 20 m-matched children with ASD, and the 26 m TD children and 26 m-matched children with ASD, were matched on PCDI scores, the expectation would be that their word order usage would also be similar, and the analyses bore this out for the most part; most comparisons were not significant. However, the 20 m-matched children with ASD produced more Multiverb utterances, *t*(19) = −2.268, *p* = 0.046, and a higher percentage of utterances in the OV frame than the 20 m old TD children, *t*(19) = −2.773, *p* = 0.016. Also, unexpectedly, the 26 m TD children used more SVt frames, *t*(19) = 2.703; *p* = 0.014, and significantly higher percentage of utterances in (Loc)You(NP) frames than the 26 m-matched children with ASD, *t*(19) = 3.096, *p* = 0.008. In contrast, the 26 m-matched children with ASD produced a significantly greater percentage of utterances in the VtO word order compared to their TD peers, *t*(19) = −4.063, *p* = 0.001.

Table 4 compares the caregivers’ and children’s percentages of word order utterances. Whereas caregivers produced significantly higher proportions of the SVi, SVt, SVtO, OV, and Multiverb orders than their 20-month old TD children did, the other word orders were produced at similar – albeit low – proportions for both 20 m TD children and their caregivers. Caregivers only produced two frames at significantly higher proportions than their 26-month-old TD children did; namely, the SVt and (Loc)You(NP) frames. Additionally, these children produced the (S)zai(Loc) order at higher proportions than their caregivers did. Interestingly, the proportions of word order usage between caregivers and children were more similar within the ASD groups, with caregivers producing marginally higher proportions of the (S)zai(Loc) and (Loc)You(NP) frames, compared to the 20 m-matched children with ASD, and significantly higher proportions of the SVt order than the 26 m-matched children with ASD. The VtO order was produced at higher proportions by 26 m-matched children with ASD than by their caregivers.

**Table 4:** Children’s word orders uses compared to caregivers by diagnostic groups.

Word order	Caregivers (TD)% Mean (*SD*)	Children (TD)% Mean (*SD*)	*t*	*p*	Caregivers (ASD)% Mean (*SD*)	Children (ASD)% Mean (*SD*)	*t*	*p*
***20 m***
SVi	4.44 (2.7)	2.04 (3.0)	−1.876	0.077	**2.60 (1.7)**	**1.50 (2.6)**	**−1.155**	**0.262**
SVt	9.17 (1.7)	3.44 (4.9)	−3.506	0.005	**9.57 (5.8)**	**9.04 (19.3)**	**−0.086**	**0.932**
SVtO	7.44 (2.7)	2.08 (4.1)	−3.435	0.003	**4.33 (1.7)**	**5.78 (11.9)**	**0.398**	**0.695**
**VtO**	**7.72 (3.1)**	**6.81 (7.4)**	**−0.361**	**0.724**	**6.91 (1.8)**	**6.55 (5.9)**	**−0.190**	**0.853**
OV	1.28 (1.1)	0.10 (0.3)	−3.258	0.008	**1.68 (0.7)**	**1.85 (2.5)**	**0.219**	**0.831**
**(S)Pnom**	**6.22 (2.8)**	**9.85 (20.5)**	**0.554**	**0.586**	**8.99 (4.9)**	**7.91 (14.5)**	**−0.235**	**0.817**
**(S)zai(Loc)**	**0.87 (0.6)**	**0.75 (1.6)**	**0.224**	**0.825**	0.97 (1.0)	0.29 (0.6)	−1.926	0.068
**(Loc)You(NP)**	**3.39 (1.9)**	**6.23 (12.1)**	**0.731**	**0.474**	2.62 (1.7)	0.88 (2.4)	−1.956	0.065
Multiverb	19.31 (5.8)	1.95 (3.2)	−8.284	<0.001	**13.53 (4.9)**	**10.41 (9.5)**	**−0.968**	**0.348**
***26 m***
**SVi**	**4.0 (1.77)**	**4.58 (3.5)**	**0.466**	**0.647**	**3.69 (2.0)**	**3.07 (3.3)**	**−0.528**	**0.603**
SVt	11.67 (4.3)	7.95 (1.9)	−2.491	0.028	7.66 (2.2)	4.31 (3.8)	−2.519	0.020
**SVtO**	**7.25 (2.7)**	**5.33 (5.0)**	**−1.078**	**0.295**	**5.54 (2.4)**	**4.11 (3.8)**	**−1.060**	**0.302**
**VtO**	**6.07 (2.5)**	**7.57 (4.5)**	**0.922**	**0.369**	6.70 (1.9)	15.62 (4.5)	5.982	<0.001
**OV**	**0.97 (0.5)**	**1.61 (1.4)**	**1.329**	**0.211**	**1.88 (1.0)**	**2.73 (3.2)**	**0.857**	**0.847**
**(S)Pnom**	**8.07 (2.9)**	**6.63 (9.2)**	**−0.470**	**0.644**	**10.43 (3.2)**	**7.90 (7.0)**	**−1.092**	**0.293**
(S)zai(Loc)	1.10 (0.7)	2.87 (2.5)	2.147	0.057	**1.27 (1.1)**	**2.26 (3.5)**	**0.893**	**0.382**
(Loc)You(NP)	3.95 (1.6)	8.81 (5.0)	2.944	0.013	**3.73 (1.7)**	**3.42 (2.5)**	**0.334**	**0.742**
**Multiverb**	**18.53 (4.9)**	**14.37 (9.5)**	**−1.229**	**0.240**	**16.20 (3.1)**	**14.07 (6.9)**	**−0.903**	**0.382**

Data in bold refer to children’s word order usages, which were not significant different from those of caregivers.

We next assessed the degree of productivity with which the children used their word orders; namely, the degree to which the frames were generalized rather than rote or stereotyped. Many of the percentages in Table 3 are quite low; that is, for some of these word orders, each child produced only 1–4 utterances, so degree of productivity was impossible to determine. However, for some of the higher-frequency frames, estimates of productivity were feasible. We first explored the productivity of the SVt and VtO frames for the 26-month-old TD and 26 m-matched children with ASD, by calculating the type/token ratios and frequencies of imitation from the previous caregiver utterance for each frame. The type-token ratios [i.e., the number of types of SVt (*you look, he play*, and *I like*) divided by the total number of SVt utterances] reveal the degree to which the exact same utterance of a frame is used multiple times; higher TTRs indicate a greater number of different instances of the same frame, hence greater productivity. The imitation frequencies were calculated by comparing the number of child utterances of a given frame that were exact imitations of immediately previously produced caregiver utterances. The results, presented in Table 5, indicate that both groups show high TTRs for both frames. Moreover, there is no significant group difference in the TTR for either frame. Furthermore, the percent of VtO and SVt utterances that were imitated from immediately preceding caregiver speech is low for both groups and both frames, suggesting that the children’s utterances were not direct imitations. These findings suggest that the VtO and SVt word orders produced by the children with ASD were not stereotyped, nor were they a simple mirror of their input.

**Table 5:** SVt and VtO used by 26 m TD children and 26 m-matched children with autism spectrum disorder (ASD).

	26 mTD Mean (*SD*)	26 m-matched ASD Mean (*SD*)	*t*	*p*
**SVt**
Type/token ratio(%) Imitated SVt(%)	92.22(13.9) 0.00(0)	70.78(40.7) 10.53(20.1)	1.644 −1.739	0.125 0.113
**VtO**
Type/token ratio (%) Imitated VtO(%)	89.36(12.4) 3.42(5.9)	85.51(10.6) 9.10(20.6)	0.768 −0.840	0.452 0.412

Our second consideration of productivity focused on the Multiverb utterances of the 20-month-old TD and 20 m-matched children with ASD, because of the surprising finding that the 20 m-matched children with ASD produced more Multiverb utterances than the 20 m TD children did. Tables 6, 7 list the subtypes of Multiverb utterances used by both groups. Only four of the 10 TD children produced any Multiverb sentences at all, with two children (GZ, DD) and one frame (V1V2) comprising most of these utterances. Seven of the 11 children with ASD produced at least one Multiverb utterance, with three children (HPP, ZQ, and LMJ) and two frames (SV1V2, V1V2) comprising most of these utterances. Scrutiny of the transcripts revealed that these Multiverb utterances were not immediate imitations of caregiver utterances; therefore, we next examined whether these utterances could be considered formulaic vs. productive. A large percentage of the Multiverb utterances produced by both TD children (*M* = 70%, range = 50–100%) and children with ASD (*M* = 75.16%, range = 52–100%) were formulaic (see Appendix A). Most of the sentences were in form of “*S + yao4 + V2(O)* …”(*S* + want+*V2O*), especially *“wo3 (bu2) yao4 chi1”*(I want/not want to eat) which is a routine that the dyads frequently engaged in. Thus, although 20 m-matched children with ASD produced significantly more Multiverb frames than 20 m TD children, these were not indicators of more advanced syntax because so many of their Multiverb utterances were formulaic, and so likely not productive.

**Table 6:** Frequency of Multiverb sentences used by 20 m TD children.

	XWZ	DD	JJ	GZ	Additional children (6)	Total
V1V2	2	6		3	0	11
V1NV2		1	1		0	2
SV1V2				1	0	1
V1POV2		1			0	1
YouNV				1	0	1
Total	2	8	1	5	0	16

**Table 7:** Frequency of Multiverb sentences used by 20 m-matched children with ASD.

	HHD	YLL	LMJ	ZQ	TJN	XML	HPP	Additional children (4)	Total
SV1V2		1	5	7	2	5	19	0	39
V1V2	3		7	9	5	5	1	0	30
SV1V2O				9			4	0	13
V1V2O			5	5	1			0	11
V1OV2			3					0	3
OV1V2				3				0	3
SV1V2V3				2			1	0	3
SV1V2OV3			1	1				0	2
SV1POV2							2	0	2
V1OV2	1							0	1
SVPnom			1					0	1
V1BaOV2			1					0	1
V1SV2O			1					0	1
YouNPV			1					0	1
OV1V2V3				1				0	1
S1V1S2POV2				1				0	1
SV1BaOV2				1				0	1
V1O1BaO2V2				1				0	1
V1V2V3					1			0	1
SV1OV2						1		0	1
Total	4	1	25	40	9	11	27	0	117

### Caregivers’ Word Order Input

Caregivers’ usage of each frame for each age/language-level group is presented in Table 8. There were no significant differences in the caregiver word order usage of TD children across ages, i.e., no age-related differences in the input of caregivers of TD children. Within the ASD group, the only age-related difference in input was with the total utterance measure, where the caregivers of the 26-month matched children with ASD produced more utterances than the caregivers of the 20-month matched children, *t*(20) = −3.032, *p* = 0.007. Table 8 also displays the comparisons across diagnostic groups, with caregivers of 20-month TD children producing a significantly higher percentage of their speech as SVtO and Multiverb utterances, as well as produced significantly more total utterances, compared with caregivers of 20 m-matched children with ASD. In contrast, caregivers of these children with ASD produced a significantly higher percentage of their speech in the single verb Vt frame than caregivers of 20-month-old TD children. At the 26-month level, caregivers of TD children produced a significantly greater percentage of their utterances as SVt word orders compared to caregivers of children with ASD, who used a greater percentage of OV utterances than caregivers of TD children did.

**Table 8:** Between-group comparison of word order utterances (Caregiver; percent of utterances).

Word order	Caregivers of 20 m TD Mean (*SD*)	Caregivers of 20 m-matched ASD Mean (*SD*)	*t*	*p*	Caregivers of 26 m TD Mean (*SD*)	Caregivers of 26 m-matched ASD Mean (*SD*)	*t*	*p*
Vi	10.81(4.9)	13.79(6.3)	−1.210	0.241	9.23(3.9)	12.80(4.1)	−2.028	0.057
**Vt**	**13.83(3.4)**	**21.13(8.7)**	**−2.568**	**0.023**	13.68(6.1)	15.93(5.2)	−0.912	0.373
Va	2.20(0.8)	3.12(2.4)	−1.190	0.256	2.02(0.8)	2.15(2.0)	−0.197	0.846
SVi	4.44(2.7)	2.60(1.7)	1.883	0.075	4.0(1.77)	3.69(2.0)	0.364	0.720
**SVt**	9.17(1.7)	9.57(5.8)	−0.215	0.833	**11.67(4.3)**	**7.66(2.2)**	**2.722**	**0.014**
**SVtO**	**7.44(2.7)**	**4.33(1.7)**	**3.141**	**0.005**	7.25(2.7)	5.54(2.4)	1.568	0.133
VtO	7.72(3.1)	6.91(1.8)	0.747	0.464	6.07(2.5)	6.70(1.9)	−0.641	0.529
OV	1.28(1.1)	1.68(0.7)	−0.992	0.334	**0.26(0.4)**	**1.88(1.0)**	**−2.701**	**0.017**
(S)Pnom	6.22(2.8)	8.99(4.9)	−1.570	0.133	8.07(2.9)	10.43(3.2)	−1.758	0.095
(S)zai(Loc)	0.87(0.6)	0.97(1.0)	−0.278	0.784	1.10(0.7)	1.27(1.1)	−0.439	0.666
(Loc)You(NP)	3.39(1.9)	2.62(1.7)	0.970	0.344	3.95(1.6)	3.73(1.7)	0.307	0.762
**Multiverb**	**19.32(5.8)**	**13.53(4.9)**	**2.488**	**0.022**	18.53(4.9)	16.20(3.1)	1.321	0.202
**Total utterances**	**505.70(140.8)**	**390.45(108.3)**	**2.114**	**0.048**	476.90(112.7)	548.64(134.9)	−1.316	0.204
MLU	3.55(0.5)	3.41(0.8)	0.459	0.651	3.87(0.6)	3.71(0.6)	0.609	0.550

Data in bold refer to comparisons of word order usages which reached significance.

### Relationships Between Children’s Speech and Caregiver Input

In total, we calculated correlations between the percent usage of 12 word orders [Vi, Vt, Va, SVi, SVt, SVtO, VtO, OV, (S)Pnom, (S)zai(Loc), (Loc)You(NP), and Multiverb utterance], as well as total utterances and MLU, of the children and their caregivers. The correlations are presented in Table 9.

**Table 9:** Correlations of word order uses between caregivers and children (*r* values, *p* values).

	TD20 m	TD26 m	2 TD groups	ASD20 m	ASD26 m	2 ASD groups
Vi	0.300 (*p* = 0.400)	0.386 (*p* = 0.271)	0.349 (*p* = 0.132)	−0.016 (*p* = 0.964)	−0.122 (*p* = 0.721)	−0.042 (*p* = 0.852)
Vt	−0.187 (*p* = 0.605)	**0.721** ^*^ **(*p* = 0.019)**	0.275 (*p* = 0.241)	0.023 (*p* = 0.945)	0.477 (*p* = 0.138)	0.174 (*p* = 0.438)
Va	−0.013 (*p* = 0.971)	0.325 (*p* = 0.360)	0.112 (*p* = 0.640)	−0.069 (*p* = 0.841)	0.324 (*p* = 0.331)	0.062 (*p* = 0.785)
SVi	0.223 (*p* = 0.536)	**0.672** ^*^ **(*p* = 0.033)**	0.332 (*p* = 0.153)	0.363 (*p* = 0.273)	0.261 (*P* = 0.438)	0.356 (*p* = 0.104)
SVt	0.064 (*p* = 0.862)	0.188 (*p* = 0.602)	0.268 (*p* = 0.254)	−0.250 (*p* = 0.458)	0.475 (*p* = 0.139)	−0.151 (*p* = 0.504)
SVtO	−0.243 (*p* = 0.499)	0.392 (*p* = 0.262)	0.080 (*p* = 0.737)	0.291 (*p* = 0.386)	0.202 (*p* = 0.551)	0.175 (*p* = 0.436)
VtO	0.419 (*p* = 0.228)	0.150 (*p* = 0.678)	0.292 (*p* = 0.212)	−0.066 (*p* = 0.847)	0.393 (*p* = 0.232)	0.063 (*p* = 0.782)
OV	−0.307 (*p* = 0.388)	**0.416** ^**^ **(*p* = 0.006)**	0.072 (*p* = 0.762)	0.418 (*p* = 0.200)	0.509 (*p* = 0.110)	**0.485** ^*^ **(*p* = 0.022)**
(S)Pnom	−0.353 (*p* = 0.317)	**0.832** ^**^ **(*p* = 0.003)**	0.048 (*p* = 0.841)	0.512 (*p* = 0.108)	−0.147 (*p* = 0.665)	0.344 (*p* = 0.117)
(S)zai(Loc)	0.369 (*p* = 0.295)	0.378 (*p* = 0.282)	0.411 (*p* = 0.072)	−0.259 (*p* = 0.443)	0.079 (*p* = 0.817)	0.083 (*p* = 0.715)
(Loc)You(NP)	**0.879** ^**^ **(*p* = 0.001)**	0.285 (*p* = 0.425)	**0.702** ^**^ **(*p* = 0.001)**	−0.189 (*p* = 0.578)	0.046 (*p* = 0.893)	0.097 (*p* = 0.667)
Multiverb	0.253 (*p* = 0.480)	0.330 (*p* = 0.352)	0.140 (*p* = 0.555)	0.568 (*p* = 0.068)	0.155 (*p* = 0.650)	**0.475** ^*^ **(*p* = 0.026)**
MLU	0.216 (*p* = 0.548)	0.550 (*p* = 0.125)	**0.503** ^*^ **(*p* = 0.028)**	0.558 (*p* = 0.074)	**0.743** ^**^ **(*p* = 0.009)**	**0.638** ^**^ **(*p* = 0.001)**

* *p* < 0.05;

** *p* < 0.01.

**Figure 1:**
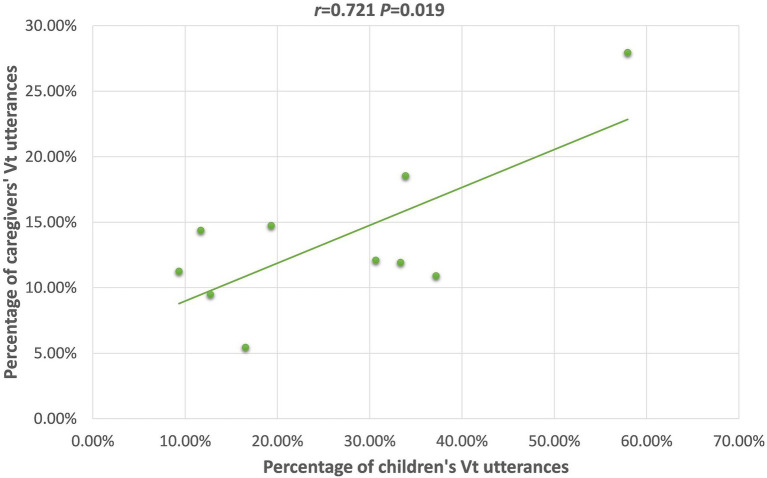
Scatterplot of 26 m TD children’s and caregivers’ Vt utterances.

**Figure 2:**
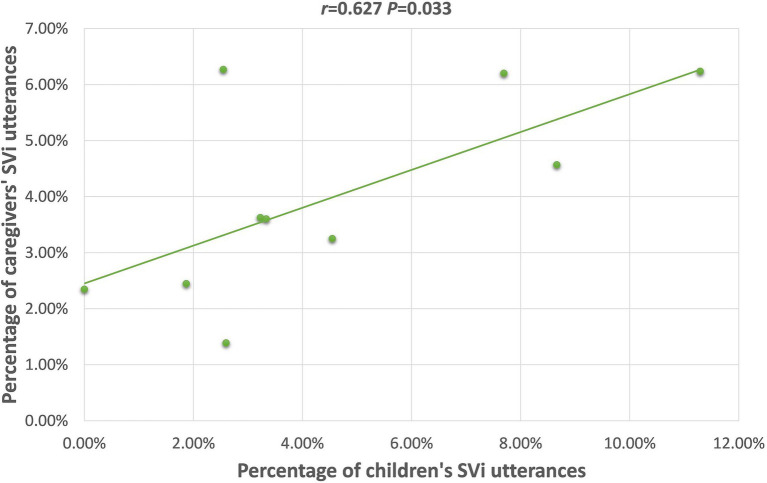
Scatterplot of 26 m TD children’s and caregivers’ SVi utterances.

**Figure 3:**
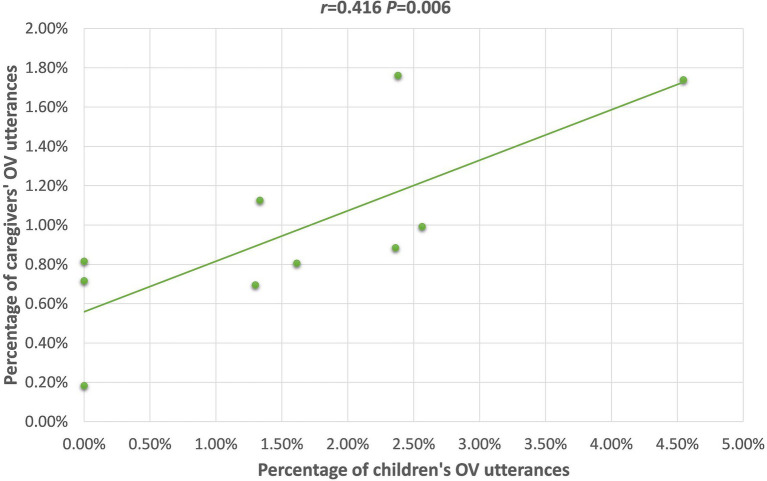
Scatterplot of 26 m TD children’s and caregivers’ OV utterances.

For the TD group, caregivers’ uses of Vt, SVi, OV, (S)Pnom, and (Loc)You(NP), were positively correlated with the children’s use of these frames. Scrutiny of the scatterplot of each relationship revealed, though, that the correlations between caregiver and child frequency of producing the (Loc)You(NP), and (S)Pnom word orders were likely only significant because of 1–2 outlier children; therefore, these will not be further discussed. The scatterplots for the relationships with the Vt, SVi, and OV frames are presented in Figures 1–3; for these, the relationships appear to be stable across the dataset, not appearing to rely on outliers for their significance.

**Figure 4:**
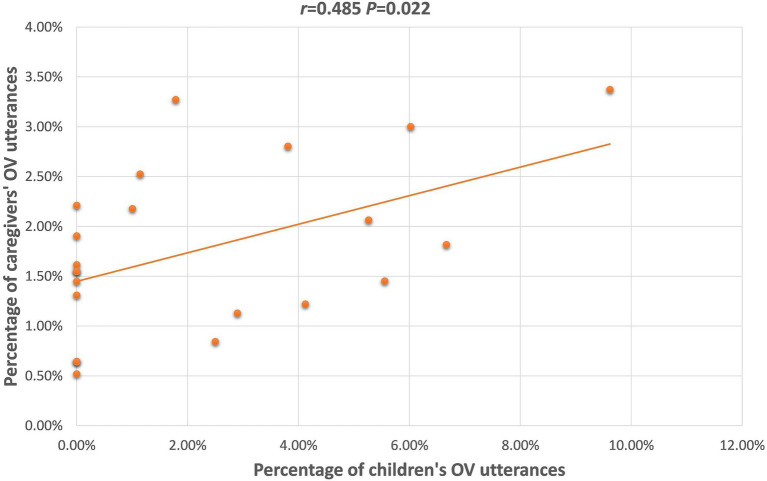
Scatterplot of children with ASD’s and caregivers’ OV utterances, combined across language.

**Figure 5:**
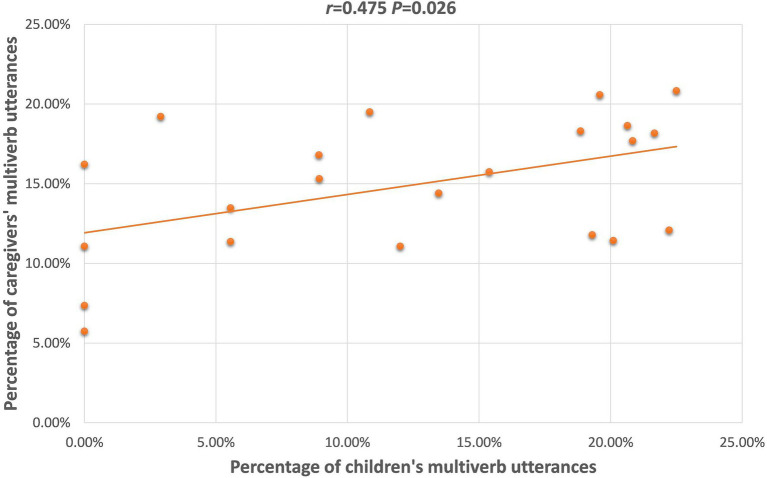
Scatterplot of children with ASD’s and caregivers’ Multiverb utterances, combined across.

**Figure 6:**
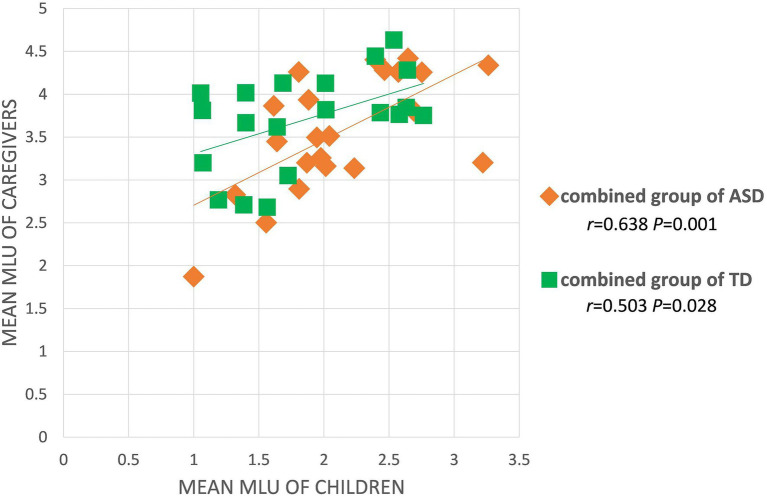
Scatterplot of MLUs between caregivers and the combined group of TD children or children with ASD.

For the ASD group, caregivers’ uses of OV, and Multiverb utterances were positively correlated with those of the children. Figures 4, 5 depict the significant relationships between caregivers and both groups combined of children with ASD in production of the OV frame, and of Multiverb utterances. Finally, Figure 6 illustrates the strong positive relationships involving MLU, between caregivers and the combined groups of TD children, and the combined groups of children with ASD.

These latter relationships between caregiver and child MLU warrant closer investigation, because of the attested relationships between caregiver and child Multiverb utterances, and because it is likely the case that caregivers who produced more Multiverb frames probably also produced longer sentences. Thus, we performed partial correlations to explore whether the child-caregiver correlations for Multiverb utterances and MLU are actually based on the shared variance. Two partial correlations were performed, one between children’s and caregivers’ Multiverb utterances, controlling for caregivers’ MLU, and the other between children’s and caregivers’ MLU, controlling for caregivers’ Multiverb utterances. For the ASD group, neither of the correlations remained significant after this control (*r*s < 0.3); however, for the TD group, the correlation between caregiver and child MLU, controlling for caregiver Multiverb utterances, remained significant (*r* = 0.509, *p* < 0.05).

## Discussion

In this paper, we explored the Mandarin grammatical usage of 20 and 26-month-old TD children and of language-matched children with ASD, with a focus on word order production during caregiver-child interactions. Comparisons of word order usage were carried out between children across ages/language levels, between TD children and children with ASD, between caregivers and children, between caregivers of the two age groups in TD group and the two language groups in ASD group, and between caregivers of TD children and those of children with ASD. Moreover, children’s productivity of the most frequently used word orders was also examined. Finally, correlations were carried out between caregivers’ and children’s usage of the same frames. The major findings are as follows:

1. Mandarin-speaking TD toddlers began to produce multi-word sentences with subjects and objects as early as 20 months of age and used most of the multi-word frames during their 3rd year of life; moreover, contrary to our hypothesis, the language-matched children with ASD performed very similarly, producing SVt and VtO utterances at earlier ages (TD)/lower language levels (ASD) than SVtO utterances. Significant differences between the language-matched groups were rare, limited to the 20 m-matched children with ASD using more Multiverb utterances than the 20 m TD children, the 26 m-matched children with ASD producing significantly lower percentages of SVt than the 26 m TD children, who also produced significantly lower percentages of VtO than the 26 m-matched children with ASD.
2. For TD children, most SVO-related and non-canonical word orders were produced in similar proportions as their caregivers by the age of 26 months, with the VtO order reaching caregiver levels before the SVt. Similarly, for children with ASD, most SVO-related and non-canonical word orders were produced at equivalent proportions of usage as their caregivers, with only two exceptions: the 26 m-matched children with ASD used the SVt frame at lower proportions, and the VtO frame at higher proportions of their utterances, compared to their caregivers.
3. Many of the word orders were produced at frequencies too low to assess productivity; however, the SVt and VtO orders were produced frequently enough by the two older groups to allow this assessment, and both word orders were found to be productive in both groups; that is, neither stereotyped nor imitated. In contrast, a large percentage of the Multiverb utterances produced by both TD children and children with ASD were formulaic.
4. No child-age-related differences were observed in the word order usage of the caregivers of the TD children. Within the ASD group, the one language level/age-related difference in word order usage was that the caregivers of 26 m-matched children with ASD produced more utterances than those of the 20 m-matched caregivers of children with ASD. Caregiver word order usage differed somewhat between diagnostic groups, with caregivers of children with ASD producing fewer total utterances, as well as fewer SVtO, SVt, and Multiverb utterances than caregivers of language-matched TD children, who correspondingly produced fewer Vt and OV utterances.
5. Strong positive relationships were found between caregivers’ MLU and the concurrent MLU of both TD children and children with ASD. Moreover, TD children’s percent usage of the Vt, SVi, and OV orders correlated positively with that of their caregivers, as did children with ASD’s percent usage of OV and Multiverb frames. In what follows, we discuss these findings with respect to our hypotheses and the current literature.

### Successful Acquisition of Mandarin Word Orders With Ellipsis

The TD children in our sample produced SVO-related word orders such as SV and VO early at 20 months, with VO more frequent than SV. This replicated findings of Erbaugh (1982) and Kim (2000) that SV and VO were the earliest two-word combinations produced by children aged between 2 and 3;5, and that VO was used more than SV; it is noteworthy that the children in this study were even several months younger than those in previous studies. The SVtO frame was produced at adult proportions at 26 m, which is consistent with finding of Yeh (2015). The significant increase of SVt usage in 26 m TD learners compared with 20 m TD children is consistent with studies of Wang et al. (1992) and Kim (2000), demonstrating that as the MLUs of Mandarin-speaking children increased, the mean percentage of their sentences with null subjects decreased, while the mean percentage of their sentences with null objects increased. Thus, they appear to be able to appropriately omit subjects and/or objects. One reason for the little change in VtO utterances between 20 and 26 m TD children in our study might be that the age gap is much smaller (6 months) than that of in Wang et al.’s study (2 years). Our findings also highlight how TD children produced non-canonical word orders, including OV, (S)Pnom, (Loc)You(NP), and (S)zai(Loc) as early as 20 and 26 months of age. With our small sample size and large SDs, it is hard to determine whether they have reached adult levels of these non-canonical orders; however, the TD children in our study appear to be producing non-canonical word orders earlier and more frequently than those in Yeh (2015).

Similar to the language-matched TD children, the children with ASD in our study showed the ability to use Mandarin grammatical structures with omitted subjects and objects, despite their pragmatic deficits. This was contrary to our prediction that children with ASD would produce SVtO word orders more frequently than SVt and VtO orders because of hypothesized difficulties with knowing when subjects and objects could be appropriately omitted. It might be possible that these early SVt and VtO utterances were intended by the children to be produced as SVtO, with the omission occurring because of a production-length limitation (Gerken, 1990). However, we find this unlikely because these children produced the VtO and SVt orders productively; i.e., with multiple verbs and nouns. Moreover, a simple length limit would have arguably resulted in many more SVt utterances than VtO utterances, whereas we observed numerous occurrences of both.

Interestingly, 26 m-matched children with ASD used VtO frames significantly more frequently than 20 m-matched children with ASD, and they produced significantly more VtO and fewer SVt than 26 m TD children (Table 3). Their use of the VtO frame was also a much greater proportion of their utterances, compared to their caregivers (Table 4). It seems that when moving to the two-word or multi-word stage, 26 m TD children tended to combine subjects with verbs, while children with ASD preferred to produce verbs with objects. We conjecture that this under-use of SVt and over-reliance on the VtO frame in Mandarin-exposed children with ASD may be related to the observed under-use of pronouns in children with ASD learning other languages, which is likely attributable to their pragmatic deficits (Terzi et al., 2017). Previous research has shown that adult speakers tend to use more pronouns in subject position than in non-subject position (Arnold, 2008). And studies have reported larger pronoun ratio in subject position than in object position for TD children, with much more full nouns than pronouns in object position (Novogrodsky and Edelson, 2016; Terzi et al., 2017, 2019). Therefore, the more frequent omission of subjects and over use of objects in children with ASD in our study can be taken as evidence that they use a strategy to avoid pronoun uses, as researchers have reported that compared with TD children, children with ASD seems to be less sure about the pronominal use (Naigles et al., 2016; Terzi et al., 2019; Kelty-Stephen et al., 2020). Another possible interpretation for this finding, though, could be that children with ASD were less likely to talk about items and people not physically or temporally present (Eigsti et al., 2007; Su et al., 2018). Subjects in the current context were almost always children and their caregivers; thus, were more likely to be omitted (Erbaugh, 1982).

Though matched on language level, the growth in language between the 20 and 26-month-old TD children appeared more extensive than that between the 20 and 26 m-matched children with ASD. That is, compared with the 20 month TD group, 26 month TD children produced significantly more advanced grammar in terms of both quantity (total utterance) and quality (simple and complex word orders, MLU), whereas the 26 m-matched children with ASD only exceeded the 20 m-matched children with ASD in two word orders [VtO, (Loc)You(NP)]. The smaller difference between 20 and 26 m-matched children with ASD could be indicative of slower language growth in ASD overall, which has been reported for English-exposed children in the study of Tek et al. (2014). However, the large variability in the ASD groups also likely played a role. For example, the OV frame was used more frequently in the 26 m TD group compared to the 20 m TD group as well as in the 26 m-matched ASD group compared to the 20 m-matched ASD group. The average increases were similar for both groups (TD: 1.5%, ASD: 0.9%), but the variance was more than twice as high for the ASD group (see Table 3), such that the 20–26 m difference only reached significance for the TD group. In the future, larger samples are in need to decrease the variance and make the comparisons more reliable.

### Different Degrees of Productivity in Different Word Order Structures

Both 26 m-matched children with ASD and 26 m TD children generally produced their VtO word orders productively, rather than relying on stereotyped or imitated utterances (Table 5). This finding was inconsistent with our hypothesis, which was based on reports that English-speaking children with ASD produced significantly more formulas and significantly less productive language than TD children (Tager-Flusberg and Calkins, 1990). Whereas it is consistent with the finding that formulaic language might not be a major characteristic of the entire ASD spectrum (Van Lancker Sidtis, 2012; see also Chin et al., 2018). Thus, grammatical productivity, which is a major hallmark of human languages (Chomsky, 1959), seems to be preserved in at least some children with ASD.

In contrast, whereas 20 m-matched children with ASD produced more Multiverb utterances than 20 m TD children, these utterances were not generally productive, because they are routines that caregivers and children frequently engaged in; a large portion of these (70–75% of Multiverb utterances for 20 m TD children and 20 m-matched children with ASD) were captured by the fixed form “(S) + yao4 + V2(O).” Interestingly, closer scrutiny indicates that the percentage of formulaic utterances varied according to the language level of the children with ASD (see Appendix A). That is, the two children (LMJ, ZQ) with the greatest variety of Multiverb utterances and fewest formulaic frames were the ones with highest PCDI scores. These two children may be considered to be more productive in their Multiverb frame usage. The children who had not produced any Multiverb frames at all, or who produced more formulaic Multiverb utterances, had lower vocabulary scores on the PCDI. These findings are consistent with the claim that formulaic language use differs across the autism spectrum and that persons with higher expressive language produce less formulaic utterances (Van Lancker Sidtis, 2012).

### Caregiver Input Broadly Rather Than Finely Tuned to Child Speech

If caregivers are sensitive to their children’s linguistic development, then their speech would be finely tuned to children’s age and/or growing linguistic competence, which is known as the fine-tuning hypothesis (Cross, 1977; Sokolov, 1993). This study tests this hypothesis through exploring whether there are age-related changes in word order uses by caregivers of both TD children and children with ASD. The results showed no age-related changes in the word order usage of caregivers of TD children, and very few (i.e., only an increase in total utterances) for caregivers of children with ASD. In line with previous findings on Mandarin word order learning, more diverse word orders are not observed in caregivers of older children or children with higher language levels compared to those of younger children/children with lower language levels (Erbaugh, 1982; Wang et al., 1992; Yeh, 2015). Diversity in word order use may indeed not be a valid indicator of caregivers’ adaptation to children’s growing communicative competence.

Some degree of tuning to child speech might yet be observed, though, in the comparisons of the caregivers of the TD and ASD groups. That is, caregivers of 20 m TD children produced more SVtO utterances, total utterances, and Multiverb utterances, but fewer Vt utterances than those of 20 m-matched children with ASD. Furthermore, caregivers of 26 m TD children used more SVt utterances but fewer OV frames than those of 26 m-matched children with ASD. In general, then, caregivers of 20 m TD children produced sentences with higher quantity and complexity, compared to those of 20 m-matched children with ASD. These group input differences may be attributable to the lower level of social and communicative skill displayed by the children with ASD, though they were language-matched to the TD children. Severity of ASD symptoms may result in diminished communication and have negative impacts on caregivers’ communicative style (Kim and Mahoney, 2004; Lehr et al., 2016). Thus, the caregivers were broadly tuning their speech to their child’s social-communicative levels, but not specifically or finely to their structural language levels.

An additional reason, though, for the group differences in input may lie in group differences we observed in the caregivers’ education background and economic status, which were not controlled in this study. For example, the primary caregiver of one child (LL) in the 20 m-matched group with ASD was a grandmother, who was likely born around 1950–1960. In China, most women of this age usually have education backgrounds below middle or high school. Consistent with research from other cultures (Meredith et al., 2016) that caregivers with lower education levels speak less to their children, LL’s grandmother’s total utterances (388) and use of Multiverb utterances proportion (11.08%) were lower than the average level (390.45; 13.53%) in the 20 m-matched group of caregivers. Her usage of the SVtO frame (1.03%) was the lowest in this group (average level: 4.33%). The small sample size in this study allowed these 1–2 participants to more strongly affect the group’s profile.

Caregivers of 26 m TD children and 26 m-matched children with ASD did not vary so much by the complexity of their input; that is, the SVt frame was used more frequently by caregivers of the TD children (see example 2) whereas the OV frame was used more frequently by caregivers of the children with ASD (see example 3). One reason that caregivers of 26 m-matched children with ASD used sentences with fewer subjects could be that children with ASD were less likely to talk about items and people not physically or temporally present in spontaneous speech (Eigsti et al., 2007; Su et al., 2018). And when caregivers tried to follow children’s attention and focused more on the items and people in the immediate context, they easily omitted the subjects because of the shared common ground (Walton and Ingersoll, 2015). Furthermore, one reason the OV frame was more frequent in speech to children with ASD might have been because the OV frame can be used to refer to how the state of the object is affected by the subject’s action on it. That is, the O in both Mandarin (S)BaOV and OV frames can receive the patient role and denote how it is affected by the agent role S. The difference is that the OV frame is simpler and more direct with the omissions of S, whereas the morphosyntactic marker Ba may hinder children’s comprehension due to the vague meaning of Ba (Yeh, 2015; Zhou et al., 2017). Therefore, it is likely that the caregivers of 26 m-matched children with ASD used more OV instead of (S)BaOV to facilitate children’s comprehension and production. *Post-hoc* review of the OV utterances produced by the caregivers of children with ASD revealed that more than half of the OV frames (*M* = 55.37%, range = 20–100%) had the potential to become (S)BaOV utterances. And unsurprisingly, the correlation result of our data has confirmed the strong relationship of OV frame used by caregivers and children with ASD, *r* = 0.485, *p* = 0.022.

*Example 2* (SV frame used by a caregiver of 26 m TD in the context of “eating cookies”).

Mom: “Shui2 chi1 (bing3gan1)?”/Who eats (cookies)? SVt(O).

Child: “Wo3 chi1”/I eat. SVt

Mom: “Ma1ma chi1”/Mom eats. SVt

Chid: “Bao3bao chi1”/Baby eats. SVt.

*Example 3* (OV frame used by a caregiver of 26-month-matched ASD in the context of “playing pop-out toys”).

Dad: “Xiao3xiong2 an4 xia4qu4.”/The bear press down. OV

Dad: “San3ge4 xiao3xiong2 an4 chu1lai2.”/The three bears press out. OV

Dad: “Huang2se4 de xiao3xiong2 ning3 chu1lai2.”/The yellow bear screw out. OV.

In sum, on the one hand, caregivers of children with ASD demonstrated sensitivity to the language development of their children in that they increased the amount of total utterances as the language ability of their children increased, which is consistent with the previous findings (Bang and Nadig, 2015; Fusaroli et al., 2019). However, caregivers of children with ASD also showed little specific fine-tuning, in that there were no changes between the word order input to two groups of children with ASD at different language levels, similar to caregivers of two groups of TD children. As a language without case-marking system, Mandarin relies heavily on word order to convey the idea; however, its constraints such as pragmatic factors make Mandarin word order an inflexible choice in most situations (Zhang, 1994). For example, known information is usually placed at sentence-initial position and new information at sentence-final position in Mandarin. The variety of word order use by caregivers, therefore, is not a clear-cut indicator of more advanced language level for children with ASD. What may matter more for children’s future language development might be whether caregivers’ utterances follow up on children’s attention or not, i.e., verbal responsiveness (see So et al., 2021 for a recent investigation of caregivers following in on the attention of Chinese-speaking children with ASD).

### Strong Caregiver-Child Relationships in General Language but Fewer in Specific Aspects of Language

For both TD children and children with ASD, the caregiver input related only sparingly to the children’s word orders, which is consistent with finding of Yeh and Naigles (2014) for TD children. In that study, significant concurrent correlations were found in the frequency of usage of the Vi, Vt, Va, SVtO, and (S)Pnom orders between caregivers and 26 m TD children, with the Vt frame also strongly correlated between caregivers and 20 m TD children. Word orders with strong correlations in the current study (Vt, SVi, and OV frames between caregivers and 26 m TD children) were also few, with the Vt frame being the only replicated word order correlation between the two studies. For children with ASD, only the usage of OV and Multiverb utterances were strongly correlated between caregivers and children with ASD, with the only overlapping frame between TD children and children with ASD being the OV order. These “scattered” results should be interpreted with caution, because of course relationships between caregiver and child speech rely on there being sufficient child speech, and the children in both Yeh and Naigles (2014) and our study had not produced many of the word orders used by their caregivers (Cameron-Faulkner et al., 2003). Moreover, both studies have a small number of participants; hence the correlations might be unstable and clearly need to be replicated with larger samples.

One further reason for the spare relationships between grammatical structures of caregivers and children might be that the word orders of caregivers do not always elicit the exact same word orders from children. For example, when a mother asks her child “Ni3 yao4 bing3gan1 ma1?”(Do you want cookies?; SVtO) in Mandarin, the child would very much likely to answer “Yao4/bu2yao4”(want/do not want; Vt) or “Wo3 yao4/bu2yao4”(I want/do not want; SVt) rather than “Wo3 yao4/bu2yao4 bing3gan1”(I want/do not want cookies; SVtO). Therefore, it seems that the SVtO frame in caregivers is more likely to elicit single verb utterances or SVt utterances from children. Moreover, spare relationships in word orders between both TD children and children with ASD suggest that caregivers who produced a specific word order in most cases did not have children do the same. The data here may support the claim that while frequency clearly matters in children’s first language acquisition, it does not dominate in every aspect (Yang, 2015), such as word order learning in this study.

We suggest, as well, that there might be some innate principles which guided children’s acquisition of word orders, such as the one-to-one structural mapping principle (Fisher and Gleitman, 2002). According to this principle, children would assume that verbs in sentences with two noun phrase arguments should be mapped onto actions with two thematic roles (Lidz et al., 2001), i.e., assign agent roles to subjects and patient roles to objects. Findings with TD children have revealed that very young children (19 months in French, 17 months in Mandarin) have acquired the abstract representation of word order of their language with very limited input (Franck et al., 2013; Zhu et al., 2021), which might support the stronger role of children’s innate principles over caregiver input in word order acquisition. Therefore, possession and/or utilization of the one-to-one mapping principle might also account for the sparse correlations observed between word orders used by children and their caregivers in our study.

However, significant and positive correlations for MLU emerged in our study, between caregivers and both TD children and children with ASD, which suggest that caregivers who produced longer sentences had children who also produced longer sentences. This replicates the previous findings of English-speaking caregivers and children with ASD or TD (Frank et al., 1976; Bang and Nadig, 2015; Fusaroli et al., 2019). According to this research, longer MLUs in caregiver input may scaffold and promote children to produce longer MLUs themselves. For example, here are two episodes of caregiver-child talk about remote-controlled toy car with different MLUs.

*Example 4* (child MLU: 2.396, caregiver MLU: 4.443) 26 m TD dyad.

Dad: Wo3men na2 yao2kong4qi4 lai2 yao2kong4 yi2xia4 qi4che1, hao3bu4hao3?

/We use the remote controller (to) control the car, okay? SV1O1V2V3O3.

Child: Hao3, wo3 yao4 dai4zhe da2che1 jian3 shu4ye4.

/Okay, I want to take the car (to) pick leaves. SV1V2O2V3O3.

*Example 5* (child MLU: 1.869, caregiver MLU: 3.201) 26 m-matched ASD dyad.

Dad: Ni3 wan2 yao2kong4./You play (the) remote controller. SVtO.

Child: Yao2kong4./Remote controller.

Song and So (2021) did not find such a relationship between parent MLU and child MLU; however, their methods were different from ours, and their sample of children was much older than ours. It will, of course, be important to further investigate relationships between parent speech and child language outcomes across developmental time. Interestingly, in our study, based on the shared variance of MLU, Mandarin-speaking caregiver who produced more Multiverb utterances also produced longer sentences, and the correlation between TD children and their caregivers’ MLU was the strongest of all. In sum, this relationship between caregiver input and children speech cuts both ways – if children hear more complex (longer MLU, more Multiverb frames), they produce more complex speech, and if they hear simpler frames (Vt, SVi), they produce simpler speech.

## Limitations and Conclusion

Limitations of this study are as follows: First of all, the small sample sizes in this research, together with large SDs in children’s uses of word orders, often resulted in small effect sizes in data analysis, making comparisons and correlations less reliable. Therefore, additional participants and more age groups (such as a 32-month-old group of TD children and 32 m-matched children with ASD) will be needed to fully discover the developmental pattern of children in a more reliable way. What is more, 30-min parent-child play in lab or centers may not be the best way to elicit spontaneous speech from children as fully and naturally as possible; some children and caregivers may need more time and more familiar settings to show how they carry on daily conversations. Future research could have the dyads recorded with longer time at children’s home so as to reduce the possibility of underestimating children’s language ability (Tek et al., 2014; Naigles and Fein, 2017; Fusaroli et al., 2019). In addition, this research used cross-sectional instead of longitudinal data, which cannot fully show the relationships between caregiver input and children’s subsequent language learning because of the individual differences across age/language groups. Therefore, future work might need longitudinal data to explore the effect of caregiver input on individual word order development (Tek et al., 2014; Fusaroli et al., 2019). Finally, additional types of input features may need to be explored to account for children’s word order learning. Future analyses will also consider child and caregiver utterance types (e.g., declaratives, questions, and imperatives) as potential grammatical or pragmatic influences on word order acquisition.

We set out to examine grammatical acquisition of preschool Mandarin-exposed TD children and children with ASD who are language-matched, focusing on word orders. The productivity of the most frequently used word order frames of children was also assessed. Moreover, we examined caregivers’ utterances and finally explored relationship between caregiver input and child production. Our findings reveal that firstly, despite their pragmatic deficits, preschool Mandarin-speaking children with ASD could acquire the word orders with pervasive ellipsis of subjects/objects, which shared certain similarity with language-matched TD children; while they also displayed differences from TD children in few aspects such as the usage of SVt and VtO. Secondly, similar to TD children, word order productivity is preserved in at least some children with ASD; however, children with ASD’s Multiverb utterances were also characterized by stereotyped speech, with children who had more advanced vocabularies producing less formulaic language. Thirdly, both TD children and children with ASD experienced generally similar rates of caregivers’ input, with TD children’s input greater in amount and complexity; however, caregivers of both groups showed no evidence of fine tuning, with no age/language-related changes in word order. Lastly, caregiver input played a smaller role in children’s acquisition of specific word orders, in contrast to a bigger role in general language. That is, caregivers who produced longer/complex utterances had children who did the same. In sum, word order acquisition in Mandarin-exposed TD children and children with ASD seems to be influenced by both caregiver input and child abilities.

## Data Availability Statement

The original contributions presented in the study are included in the article/Supplementary Material, further inquiries can be directed to the corresponding author.

## Ethics Statement

The studies involving human participants were reviewed and approved by The Medical Ethics Committee of the Second Xiangya Hospital, Central South University. Written informed consent to participate in this study was provided by the participants’ legal guardian/next of kin.

## Author Contributions

YX and YS designed the study. YX carried out the experiment. YX and LN analyzed the results and wrote the manuscript. All authors contributed to the article and approved the submitted version.

## Funding

This research was sponsored by a grant from Humanities and Social Science Project of the Ministry of Education of China (No. 20YJC740074) and by China Scholarship Council (No. 201906375061), awarded to YX.

## Conflict of Interest

The authors declare that the research was conducted in the absence of any commercial or financial relationships that could be construed as a potential conflict of interest.

## Publisher’s Note

All claims expressed in this article are solely those of the authors and do not necessarily represent those of their affiliated organizations, or those of the publisher, the editors and the reviewers. Any product that may be evaluated in this article, or claim that may be made by its manufacturer, is not guaranteed or endorsed by the publisher.
