# Supplementary material for: Early Word Order Usage in Preschool Mandarin-Speaking Typical Children and Children With Autism Spectrum Disorder: Influences of Caregiver Input?
Source: Front Psychol. 2022 Jan 6;12:766133. doi: 10.3389/fpsyg.2021.766133 (PMC8770832; doi:10.3389/fpsyg.2021.766133)
Supplement: Supplementary file 1 [file Table_1.DOCX]

Appendix A

Most frequent Multiverb sentences used by 20m TD children and 20m-matched children with ASD

| Name/ total number of Multiverb used | Word order/  total number of each frame used | Frequency | Example | Translation |  |
| --- | --- | --- | --- | --- | --- |
| **20m TD children** | | | | | |
| XWZ  (n=2) | V1V2  n=2 | 1  1 | Bu2yao4 fei  Hai2 yao4 pin1 | Don’t fly  Still want (to) build | F |
| DD  (n=8) | V1V2  n=6 | 2  1 | Yao4 guan1men2  Xiang3 chi1 | Want (to) close the door  Want (to) eat | F |
|  |  | 1  1  1 | Xia4qu4 wan2  Dao4 di4shang4 wan4  Qing3 zhu4yi4 dao4che1 | Go down play  Go (to) floor play  Please mind reverse car |  |
|  | V1NV2  n=1 | 1 | Yao4 ta1 zuo4 zhe | Want him (to) sit | F |
| GZ  (n=5) | V1V2  n=3 | 1  1 | Bu2 yao4 da3  Xiang3 na2 | Don’t hit  Want (to) take | F |
|  |  | 1 | Dao4 na2li3 qu4 le? | Go where go? |  |
|  | SV1V2  n=1 | 1 | Wo3 yao4 da3 | I want (to) hit | F |
|  | YouNV  n=1 | 1 | Hai2you3 zhe4ge xia4che1 | Still there is this (to) get off the car |  |
| **20m-matched children with ASD** | | | | | |
| HHD  (n=4) | V1V2  n=3 | 2  1 | Yao4 chi1  Yao4 da3kai1 | Want (to) eat  Want (to) open | F |
|  | V1OV2  n=1 | 1 | Bang1 wo3 da3kai1 | Help me open | F |
| XML  (n=11) | V1V2  n=5 | 2  2  1 | Yao4 chi1  Bu2 yao4 chi1  Yao4 da3 | Want (to) eat  Not want (to) eat  Want (to) hit | F |
|  | SV1V2  n=5 | 5 | Wo3 yao4 chi1 | I want (to) eat | F |
| TJN  (n=9) | V1V2  n=5 | 1  3 | Xiang3 chi1  Bu4 xiang3 chi1/  kan4/an4 | Want (to) eat  Not want (to) eat/  see/press | F |
|  |  | 1 | Bu4 wan2 da3 zhao1hu1 | Not play (the game of) say hi |  |
|  | SV1V2  n=2 | 1  1 | Ni3 yao4 dai4  Shui3 yao4 chi1 | You must wear  Who want (to) eat | F |
| HPP  (n=27) | SV1V2  n=19 | 5  3  3  1  1  1  1  1  1  1 | Wo3 yao4 chi1fan4  Wo3 yao4 kai1men2  Wo3 yao4 shu1tou2/  zuo4/wan2  Wo3 yao4 fang4 jin4qu4  Wo3 yao4 pa3 shang4qu4  Wo3 yao4 xia4lai2  Ni3 yao4 xian1 zou3 le  Ni3 yao4 xi2zao3  Wo3 yao4 fang4 lan2  Jiao3jiao yao4 shui4jiao4 | I want (to) have meal  I want (to) open the door  I want (to) comb the hair/ sit down/play  I want (to) put in  I want (to) climb up  I want (to) come down  You want (to) leave first  You want (to) take a bath  I want (to) put basket  The feet want (to) sleep | F |
|  |  | 1 | Ni3 qu4 xi2zao3 | You go (and) take a bath |  |
|  | SV1V2O  n=4 | 1  1  1  1 | Wo3 yao4 xi3 wa2wa  Wo3 yao4 wan2 wa2wa  Wo3 yao4 xi3 tou2fa4  Wo3 yao4 kai1 ba1shi4 | I want (to) wash doll  I want (to) play doll  I want (to) wash hair  I want (to) drive bus | F |
|  | SV1POV2  n=2 | 1  1 | Wo3 yao4 gen1 ta1 wan2  Wo3 yao4 tong2 ta1 wan2 | I want (to) play with her  I want (to) play with her | F |
| LMJ  n=25 | V1V2  n=7 | 1  1  1  1 | Yao4 chi1  Yao4 da3qi4  Yao4 xi2zao3  Bu2yao4 qiang3 | Want (to) eat  Want (to) blow up  Want (to) take a bath  Don’t grab | F |
|  |  | 1  1  1 | Xi3huan1 chi1  Kai1shi3 xi2zao3  Chui1 bu2 dong4 | Like (to) eat  Begin (to) take a bath  Blow not move (can’t blow it away) |  |
|  | SV1V2  n=5 | 1  1  1  1  1 | Zhe4ge4 yao4 xi2zao3  Wo3 yao4 xi2zao3  Wo3 yao4 chui1 yi2xia4  Xiao3 huo3che1 yao4 chong1dian4  Wo3 yao4 qu4 xx | This needs (to) take a bath  I want (to) take a bath  I want (to) blow for a while  The little train needs charging  I want (to) go (to) xx | F |
|  | V1V2O  n=5 | 2  1  1 | Bu2yao4 da1 ji1mu4  Yao4 chi1 tang2guo3  Yao4 chi1 xiao3 man2tou2 | Not want (to) build blocks  Want (to) eat candy  Want (to) eat small buns | F |
|  |  | 1 | Yi4qi3 wan2 zuo4 dian4ti1 | Together play take elevator |  |
|  | V1OV2  n=3 | 1  1  1 | Hai2 dai4 le wan2ju4 xi2zao3  You4 zuo4 huo3che1 wan2  You4 zuo4 dian4ti1 wan2 | Also bring toys (to) take a bath  Take train (and) play again  Take elevator (and) play again |  |
|  | SV1V2OV3  n=1 | 1 | Ni3 yao4 zuo4 dian4ti1 wan2 | You want (to) take elevator (and) play |  |
|  | V1SV2O  n=1 | 1 | Xia4qu4 wo3 chi1 zao3can1 | Go down (and) I eat breakfast |  |
| ZQ  n=40 | V1V2  n=9 | 1  1  1  1 | Yao4 deng3 yi2xia4  Yao4 na2  Bu2yao4 na2  Shi4 bu2 shi4 yao4 wan2 | Need (to) wait for a while  Want (to) take  Don’t take  Is or not want (to) play | F |
|  |  | 1  1  1  1  1 | Ming2tian1 lai2 wan2  Neng2 bu4 neng2 bie2 qu4 nao4  Bie2 qu4 gao3  Bie2 qu4 dong4  Ke2yi3 you4er2yuan2 qu4 da1 | Tomorrow come play  Can no can not go (and) make (trouble)  Not go do  Not go move  Can go take (it at) kindergarten |  |
|  | SV1V2O  n=9 | 1  1  1  1  1  1 | Ni3 hai2 yao4 gan4 shen2me  Wo3 yao4 zai4 lai2 yi2 ge4  Wo3 yao4 wan2 zhe4  Wo3 yao4 wan2 ju2zi  Wo3 jiu4 yao4 wan2 tu4zi  Wo3 yao4 wan2 zhe4ge4 qi4che1 | You still want (to) do what  I want (to) do (it) again  I want (to) play this  I want (to) play orange  I just want (to) play rabbit  I want (to) play this car | F |
|  |  | 1  1  1 | Wo3 lai2 gao3 che1  Ni3 ji4de mai3 zhe4ge  Wo3 li4ke4 zuo4 xia4lai2 wan2 wan2ju4 | I come (and) play car  You remember (to) buy this  I immediately sit down (and) play toys |  |
|  | SV1V2  n=7 | 1  1  1  1  1 | Ni3 yao4 deng3 yi2xia4  Wo2 yao4 xian1 da3kai1  Wo2 yao4 fang4 jin4qu4  Wo3 ye3 yao4 chui1  Ni3 yao4 gan4ma2 | You need (to) wait  I want (to) open first  I want (to) put in  I also want (to) blow  You want (to) do what | F |
|  |  | 1  1 | Wo3 lai2 da1 yi2xia4  Ni3 guo4lai2 jiu4 guan1diao4 | I come build  Turn off (when) you come |  |
|  | V1V2O  n=5 | 1  1  1  1 | Yao4 chuan1 ku4zi  Hai2shi4 yao4 you3 man2tou2  Yao4 wan2 na4ge4 xiao3 she2  Hai2 yao4 kan4 xx | Want (to) wear pants  Still want (to) have buns  Want (to) play that little snake  Still want (to) see xx | F |
|  |  | 1 | Bie2 qu4 dong4 wo3de mo2fa3bang4 | Not go (and) take my magic wand |  |
|  | OV1V2  n=3 | 1  1 | Zhe4ge yao4 wan2  Zhe4ge yao4 yi2 yi2xia4 | This want (to) play  This need (to) move | F |
|  |  | 1 | Zhe4ge bie2 qu4 dong4 | This not go move |  |
|  | SV1V2V3  n=2 | 1  1 | Ni3 bu2yao4 lai2 bang1mang2  Wo3 yao4 lai2 da3kai1 | You don’t come (to) help  I want (to) come (and) open | F |
|  | SV1V2OV3  n=1 | 1 | Ni3 lai2 mai3 che1 qu4 | You come (and) buy car go |  |
|  | OV1V2V3  n=1 | 1 | Cha2bei1 ke2yi3 yong4lai2 he1 | Teacup can be used to drink |  |
|  | S1V1S2POV2  n=1 | 1 | Ni3 kan4 wo3 gei3 ni3 yi2 yi2xia4 | You see me move for you |  |

Note: F=Formulaic
